# Comparative Analysis of Araceae Mitochondrial Genomes: Implications for Adaptation to Ecological Transitions in Plants

**DOI:** 10.3390/genes16101241

**Published:** 2025-10-21

**Authors:** Yuxiao Chen, Shuai Gao, Jieqiong Wang, Xin Cheng, Yue Chen, Veeranjaneyulu Chinta, Shenglong Kan

**Affiliations:** 1 Marine College, Shandong University, Weihai 264209, China; qinzhen0908@163.com (Y.C.); wangjieqiong@mail.sdu.edu.cn (J.W.); 202538020@mail.sdu.edu.cn (X.C.); cyue050219@163.com (Y.C.); 2 SDU-ANU Joint Science College, Shandong University, Weihai 264209, China; 3 Shandong Aaran Nanometer Industry Development Co., Ltd., Weihai 264209, China; jay20.good@163.com; 4 Ningbo Institute of Digital Twin, Eastern Institute of Technology, Ningbo 315201, China; Chinta@idt.eitech.edu.cn

**Keywords:** mitochondrial genome, genome size variation, RNA editing, aquatic plants, evolutionary genomics

## Abstract

**Background/Objectives**: Plant mitogenomes display remarkable variation in size, structure, and gene content, yet their evolutionary causes remain unclear. Araceae, the most significant family within Alismatales, encompasses both aquatic and terrestrial lineages, providing an excellent system for studying how ecological shifts influence mitogenome evolution. **Methods**: We assembled and annotated four new mitogenomes using both short- and long-read sequencing, including three aquatic taxa (*Pistia stratiotes* L., *Spirodela intermedia* W. Koch, *Wolffia australiana* (Benth.) Hartog & Plas) and one terrestrial species (*Amorphophallus konjac* K. Koch). Along with five previously published mitogenomes, we performed comparative analyses across nine Araceae species. **Results**: These mitogenome sizes varied from ~178 kb to ~877 kb, consisting of one to 19 circular molecules, with aquatic species generally having smaller and simpler structures. Plastid-derived sequences (MTPTs) contributed 1.2–10.6% of genome content, peaking in *Zantedeschia aethiopica* (L.) Spreng. Despite significant structural heterogeneity, all species maintained core respiratory genes under strong purifying selection, while ribosomal protein-coding genes showed lineage-specific loss. RNA editing ranged from 363 to 772 sites per mitogenome, with the number of sites independent of mitogenome size. **Conclusions**: Overall, this study uncovers the dynamic evolutionary patterns of Araceae mitogenomes and offers a framework for understanding how habitat shifts between aquatic and terrestrial environments influence mitogenome diversity in plants.

## 1. Introduction

Mitochondria originated from an endosymbiotic event between proteobacteria and proto-eukaryotic cells, and over billions of years, they have evolved into the central hub of energy metabolism in eukaryotic cells [1]. In plants, however, mitogenomes (mitochondrial genomes) exhibit remarkable variation in size and structure [2]. Unlike the relatively conserved plastomes (plastid genomes), plant mitogenomes display complex architectures, including linear, branched, and multipartite forms and multiple circular molecules [2,3,4]. Frequent insertions of foreign DNA and expansions of repeats further contribute to genome sizes ranging from 66 kb to 18.99 Mb [5,6,7]. Given the central role of mitochondria in respiration, structural variations may directly affect energy metabolism, oxidative stress responses, and overall plant adaptability [8,9]. Oxygen availability, in particular, represents a critical environmental factor shaping mitochondrial function and evolution [10,11]. In land plants, aerobic respiration proceeds under relatively stable oxygen concentrations, whereas aquatic plants often experience fluctuating or hypoxic conditions, especially in submerged tissues where gas diffusion is limited. Such oxygen constraints may impose selective pressures favoring mitogenome streamlining, altered respiratory regulation, or enhanced efficiency of electron transport to maintain ATP production under low-oxygen stress. Morphological adaptations, such as adaxial stomatal localization and aerenchyma formation, further reflect these physiological adjustments [12]. Whether such differences in oxygen availability affect respiration and consequently drive the evolution of the mitogenome remains unresolved.

Araceae, the largest family within the Alismatales and the earliest to diverge within the order, encompasses approximately 3667 species across 143 genera [13,14]. Members of this family display a remarkable ecological breadth, ranging from aquatic taxa such as *Spirodela* Schleid., *Wolffia* Horkel ex Schleid., and *Lemna* L. to terrestrial taxa including *Amorphophallus*, *Colocasia*, and *Pinellia*. This ecological diversity makes Araceae an ideal system for investigating how contrasting habitats, particularly aquatic versus terrestrial environments, may shape the evolution of mitogenome structure, size, and gene content. To date, six mitogenomes have been reported in Araceae: five from terrestrial plants (*Amorphophallus albus* P. Y. Liu & J. F. Chen, *Colocasia esculenta* (L.) Schott, *Pinellia ternata* (Thunb.) Ten. ex Breitenb., *Z. aethiopica*, and *Zantedeschia odorata* P. L. Perry) and one from the aquatic lineage (*Spirodela polyrhiza* (L.) Schleid.) [15,16,17,18,19]. These mitogenomes exhibit striking variation in both organization and size. For instance, while *P. ternata*, *Z. aethiopica*, and *S. polyrhiza* each possess a single circular molecule, *Z. odorata* carries two, *C. esculenta* five, and *A. albus* as many as 19. Genome sizes span from 228,493 bp to 876,608 bp, with the aquatic *S. polyrhiza* harboring the smallest known mitogenome within the family. Beyond structural diversity, substantial variation in protein-coding gene (PCGs) content has also been reported across the Alismatales [20], with aquatic lineages frequently exhibiting extensive loss of ribosomal PCGs, suggesting potential links between habitat transitions and mitogenome reduction. Despite these intriguing findings, our current understanding of Araceae mitogenome evolution remains limited. Existing genomic data are biased toward terrestrial taxa, with only a single aquatic mitogenome sequenced [15], hindering robust comparisons between habitat types. Moreover, critical aspects such as the role of repetitive sequences and mitochondrial plastid DNA transfers (MTPTs) in driving structural variation, the extent and distribution of RNA editing events, and the selective pressures acting on respiratory PCGs have not been systematically assessed across this family [21]. A broader and more integrative comparative framework, encompassing both aquatic and terrestrial representatives, is therefore urgently needed to uncover the patterns and drivers of mitogenome evolution in this ecologically and morphologically diverse plant family.

Despite growing interest in plant mitogenome evolution, the extent to which ecological shifts have influenced mitogenome in Araceae plants remains unclear. To address this gap, the primary goal of this study was to investigate patterns of structural variation, compositions, and evolutionary dynamics in Araceae mitogenomes, with an emphasis on comparing aquatic and terrestrial lineages. We assembled and analyzed four new mitogenomes from diverse Araceae lineages, encompassing both aquatic and terrestrial habitats, including three aquatic (*W. australiana*, *S. intermedia*, and *P. stratiotes*) and one terrestrial species (*A. konjac*). Through comprehensive comparative analyses of genome size, structure, repeats, foreign sequences, RNA editing sites, and selective pressures on PCGs, our findings provide new insights into the drivers of mitogenome evolution in Araceae and a framework for investigating how distinct ecological niches between aquatic and terrestrial lineages influence mitogenome diversity.

## 2. Materials and Methods

### 2.1. Data Resource

In this study, we obtained both short-read (second-generation, Illumina, San Diego, CA, USA) and long-read (third-generation, Pacific Biosciences, Menlo Park, CA, USA) genomic sequencing data for *A. konjac*, *P. stratiotes*, *S. intermedia*, and *W. australiana* from previous studied (Table S1). Transcriptomic datasets were also retrieved for *A. konjac*, *C. esculenta*, *P. stratiotes*, *S. polyrhiza*, and *W. australiana* (Table S1), enabling the identification of RNA editing sites. Furthermore, publicly available organellar genomes from Araceae species with published mitogenomes, including *A. albus*, *C. esculenta*, *P. ternata*, *S. polyrhiza*, and *Z. aethiopica*, were incorporated for comparative analyses, along with two outgroup taxa, *Butomus umbellatus* L. and *Ruppia sinensis* Shuo Yu & Hartog (Table 1).

### 2.2. Mitogenome Assembly and Annotation

The mitogenome was assembled using a hybrid approach integrating short-read and long-read sequencing data [22]. The workflow proceeded as follows: (1) Short-read sequencing data were assessed using FastQC v0.12.1 (https://www.bioinformatics.babraham.ac.uk/projects/fastqc/, accessed on 1 September 2024), and adapter sequences, low-quality bases, and low-quality reads were removed with Trimmomatic v0.39 to generate clean reads [23]. (2) Clean reads were preliminarily assembled with the embplant_mt model in GetOrganelle v1.7.6.1 [24]. Contigs with low sequencing depth or lacking connectivity to mitochondrial contigs were filtered out using Bandage v0.9.0 [25], yielding a set of putative mitochondrial contigs. (3) Long sequencing reads were mapped to these putative mitochondrial contigs using Minimap2 v2.28 (r1209) to identify candidate mitochondrial long reads [26]. (4) Candidate long reads were assembled de novo with Flye v2.9.5 [27], and the assembly graph was examined and refined using Bandage v0.9.0 [25]. (5) The completeness of each mitogenome was further evaluated by confirming the presence of the full set of core mitochondrial PCGs. (6) The draft assemblies were polished with short-read data using Pilon v1.24 and POLCA [28,29]. (7) Genome annotation was performed using the PMGA pipeline [30], and the results were visualized with PMGmap [31].

### 2.3. Phylogenetic Reconstruction and Evolutionary Analysis

*B. umbellatus* and *R. sinensis* were selected as outgroups according to previous study [13], and phylogenetic analyses were independently conducted using on mitochondrial and plastid PCGs. Mitochondrial and plastid PCGs were extracted using TBtools v2.303 and CPStools v3.0 [32,33], respectively. Each single gene matrix was aligned using MAFFT v7.505 [34], and the resulting alignments were concatenated into a supermatrix with FASconCAT-G v1.06.1 [35]. Maximum likelihood (ML) phylogenetic reconstruction was performed with RAxML-NG under the GTR + G substitution model, and branch lengths were estimated from the ML tree [36].

Furthermore, the evolutionary rate of the concatenated mitochondrial PCGs matrix were calculated following the pipeline of Kan et al. [37]. The concatenated matrix was first converted into PAML format using EasyCodeML v1.0 [38]. Subsequently, the codeml subprogram in PAML v4.10.7 was employed (run mode = 0, codon frequency model = F3×4, model = 2) to estimate the nonsynonymous substitution rate (*d_N_*), synonymous substitution rate (*d_S_*), and selection pressure (ω = *d_N_*/*d_S_*) of mitochondrial matrix [39].

### 2.4. Repeats, Plastid-Derived Sequences and Shared DNA Analysis

We analyzed the distribution and characteristics of repeats, plastid-derived sequences, and shared DNA in nine Araceae mitogenomes. Repeats were identified using ROUSfinder.py [40]. Plastid-derived sequences within the mitogenomes were detected using BLAST v2.15.0+ with an E-value threshold of 1 × 10^−5^ and a minimum length of 50 bp [22,41]. Similarly, pairwise shared DNA among the nine mitogenomes was identified using BLAST v2.15.0+ with the same parameters [41]. Fragments exceeding 300 bp in length were subsequently visualized using the graphical module in TBtools v2.303 [32].

### 2.5. RNA Editing Site Identification

Raw RNA and DNA sequencing reads were subjected to quality assessment using FastQC v0.12.1 (https://www.bioinformatics.babraham.ac.uk/projects/fastqc/, accessed on 1 September 2024) and trimmed with Trimmomatic v0.39 to remove adapter sequences and low-quality reads [23]. RNA editing analysis was conducted only for *A. konjac*, *C*. *esculenta*, *P. stratiotes*, *S*. *polyrhiza*, and *W. australiana* (Table S1), as transcriptomic datasets were publicly available for these taxa. RNA editing sites were then identified using RES-Scanner v1.0, which was also classified editing types and quantified editing efficiencies across the five species [42].

## 3. Results

**Figure 1:**
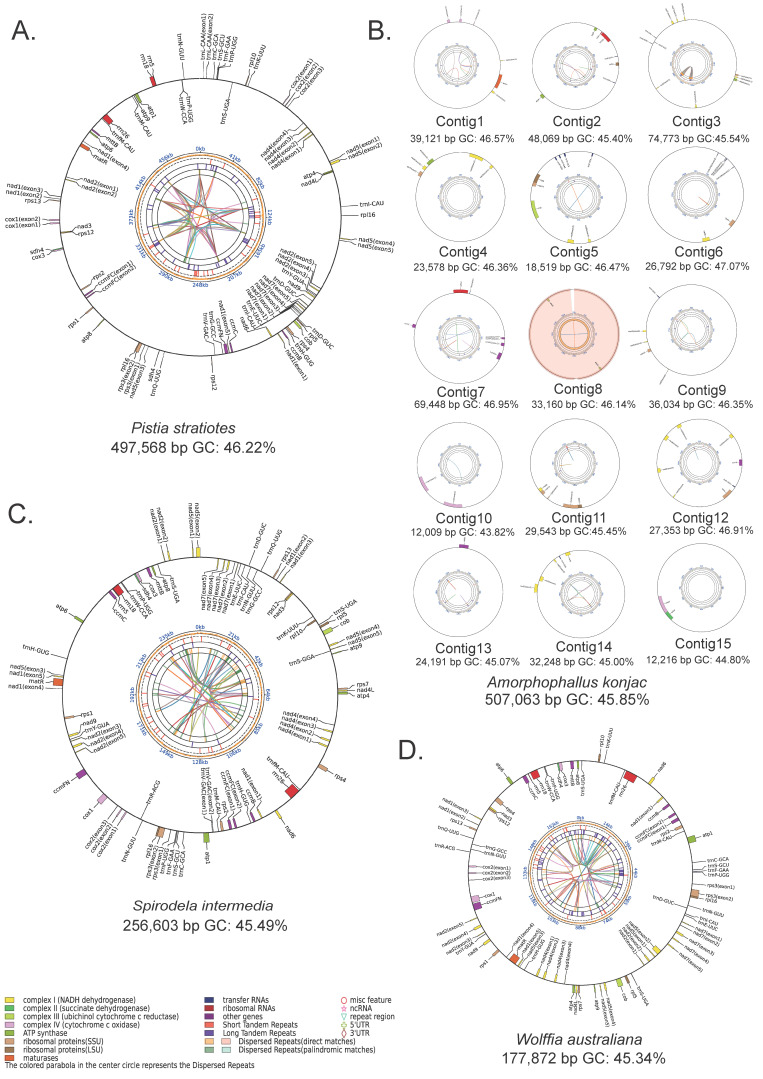
Circular maps of four newly assembled mitogenomes. (**A**) *P. stratiotes*, (**B**) *A. konjac*, (**C**) *S. intermedia*, and (**D**) *W. australiana*. From the outer to the inner tracks: gene distribution, scale, GC content, microsatellite repeats, tandem repeats, dispersed repeats, and connections between dispersed repeats. Genes are color-coded by functional category; those labeled outside the circle are encoded on the forward strand, while those inside are encoded on the reverse strand.

In this study, we newly assembled four mitogenomes from Araceae: *A*. *konjac*, *P*. *stratiotes*, *S*. *intermedia*, and *W*. *australiana* (Figure 1, Table 1). Notably, the complete mitogenomes of *Pistia* and *Wolffia* are reported here for the first time. For comparative analyses, we also incorporated five previously published Araceae mitogenomes (Table 1).

### 3.1. Gene Content Variation Among Araceae mitogenomes

All four assemblies exhibit circular structures; however, the *A. konjac* mitogenome is highly fragmented, consisting of 15 distinct circular molecules (Figure 1, Table 1). Among these species, *W. australiana* possesses the smallest mitogenome (177,872 bp), containing 36 PCGs, 21 tRNA (transfer RNA) genes, and 3 rRNA (ribosomal RNA) genes (Table 1, Tables S3 and S4). The *S. intermedia* mitogenome is slightly larger (256,603 bp) with the same set of 36 PCGs and 3 rRNA genes, but three additional tRNA genes compared to *W. australiana*. By contrast, the *P. stratiotes* mitogenome has nearly doubled in size (497,586 bp), encoding 35 PCGs, 22 tRNA genes, and 3 rRNA genes. The largest of the newly assembled genomes is *A. konjac* (507,063 bp), comprising 15 circular elements ranging from 12,009 to 74,773 bp, and encoding 55 genes in total, including 36 PCGs, 16 tRNA genes, and 3 rRNA genes.

**Figure 2:**
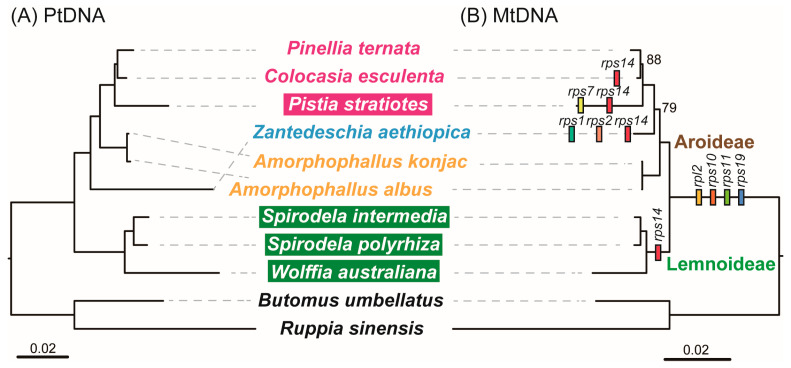
Analysis of evolutionary relationships based on chloroplast (**A**) and mitochondrial (**B**) genomes. Except for the annotations, the credibility of the rest is 100. Specie names in shade indicates aquatic taxa, the others indicate terrestrial taxa.

To investigate the evolutionary history of mitogenome in Araceae, we reconstructed phylogenies among these nine Araceae species based on plastid and mitochondrial PCGs (Figure 2). Both trees consistently divided the species into two well-supported clades corresponding to the subfamilies Lemnoideae and Aroideae. The plastid and mitochondrial trees were identical for Lemnoideae, which comprised *W. australiana*, *S. intermedia*, and *S. polyrhiza*, all with full support. In contrast, topologies within Aroideae differed between datasets. In the plastid tree, *Z*. *aethiopica* diverged first, followed by *A. albus* and *A. konjac*, and a strongly supported clade of *P. stratiotes*, *C*. *esculenta* and *P*. *ternata*. In the mitochondrial tree, however, *A. albus* and *A. konjac* diverged earliest, *Z. aethiopica* branched next (79% support), and *P. ternata*, *C. esculenta*, and *P. stratiotes* formed a clade with high to moderate support (100% and 88%).

Based on phylogenetic relationships, we traced the history of mitochondrial gene loss and transfer in the nine Araceae species (Figure 2). Four ribosomal PCGs (*rpl2*, *rps10*, *rps11*, and *rps19*) were collectively absent across all nine mitogenomes, while *rps14* was additionally lost or transferred in the Lemnoideae clade. No further gene loss was detected in *A. albus* and *A. konjac*. In contrast, *Z. aethiopica* lost *rps1*, *rps2*, and *rps14*, *P. stratiotes* lost *rps7* and *rps14*, and *C. esculenta* lost *rps14* independently. In the mitogenomes of *W. australiana*, *S. intermedia*, *S. polyrhiza* and *Z. aethiopica*, PCGs contained 23 introns, including 18 *cis*-spliced and five *trans*-spliced (Table S5). A novel *cis*-spliced intron was detected in the *cox1* gene of *A. albus*, *A. konjac*, *P. stratiotes*, *C. esculenta*, and *P. ternata*. Additionally, intron *nad1i728* of *nad1* in *P. stratiotes* and intron *rps3i74* of *rps3* in *P. ternata* exhibited a shift from *cis*- to *trans*-splicing.

### 3.2. Composition Variation Among Araceae mitogenomes

**Figure 3:**
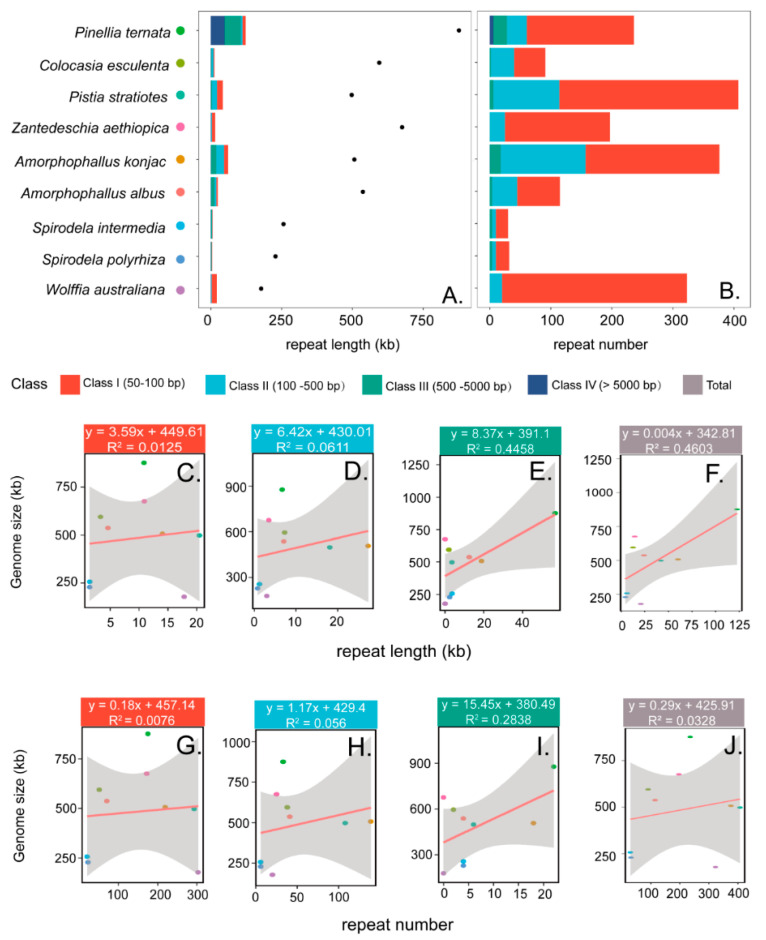
Correlation between repeats and mitogenome size. (**A**) Cumulative length of repeats in different size classes (bar plot) with mitogenome size (scatter plot). (**B**) Number of repeats in different size classes. (**C**–**F**) Relationships between cumulative repeat length of Class I (50–100 bp), Class II (100–500 bp), Class III (500–5000 bp), and Class IV (>5000 bp) and mitogenome size, respectively. (**G**–**J**) Relationships between repeat number in the four corresponding classes and mitogenome size.

Across these nine mitogenomes, genome sizes vary markedly, with *P. ternata* representing the largest (876,608 bp) and *W. australiana* the smallest (177,872 bp), reflecting an approximately five-fold size difference. According to length, dispersed repeats were classified into four categories: class I (50–100 bp), class II (100–500 bp), class III (500–5000 bp), and class IV (>5000 bp) (Figure 3). Across the nine mitogenomes, the number of repeats ranged from 30 to 407, with cumulative lengths from 4602 bp to 122,573 bp. Most species contained only class I–III repeats, whereas large repeats (>5000 bp, class IV) were detected exclusively in *P. ternata*. Class I repeats were the most abundant, ranging from 20 to 303 pairs and accounting for 56–94% of total repeats. The proportion of class II repeats varied markedly among species, from only 6% in *W. australiana* to 42% in *C. esculenta*. Class III repeats were absent from *W. australiana* and *Z. aethiopica*, but occurred in all other species, with 18 in *A. konjac*, 22 in *P. ternata*, and four in each of the remaining five species. Notably, six of Class IV repeats were identified in *P. ternata*, together accounting for 5.5% of its mitogenome length.

**Figure 4:**
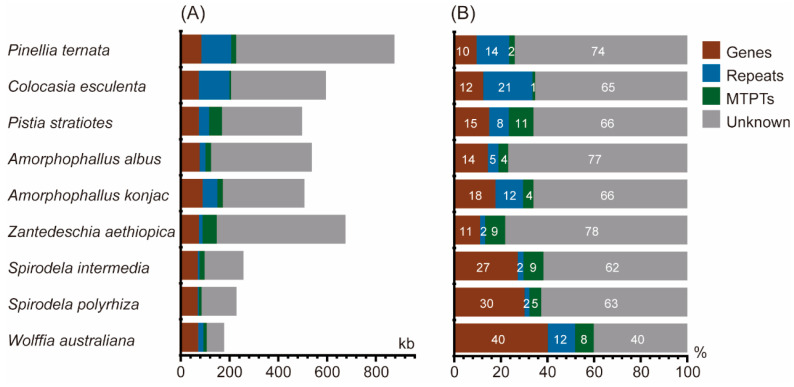
Composition of Araceae mitogenomes. (**A**) Absolute lengths of genic regions, repeats, plastid-derived sequences (MTPTs), and sequences of unknown origin relative to total genome size. (**B**) Proportional contributions of these categories to the mitogenome. “Genes” includes all protein-coding, rRNA, and tRNA genes.

Plastid-derived sequences (MTPTs) contribute between 1.19% and 10.56% of the mitogenome across the nine species (Figure 4, Table S6). The mitogenome of *C. esculenta* contains the smallest fraction, with 7.09 kb (1.19%) of plastid-derived sequences, whereas *P. stratiotes* harbors 52.53 kb (10.56%). Notably, *Z. aethiopica* contains the largest amount, with 58.27 kb of plastid-derived sequences. These results indicate marked interspecific variation in the extent of plastid-to-mitochondrion DNA transfer among *Araceae* species.

### 3.3. Shared Sequences and Sequence Collinearity Across Araceae mitogenomes

**Figure 5:**
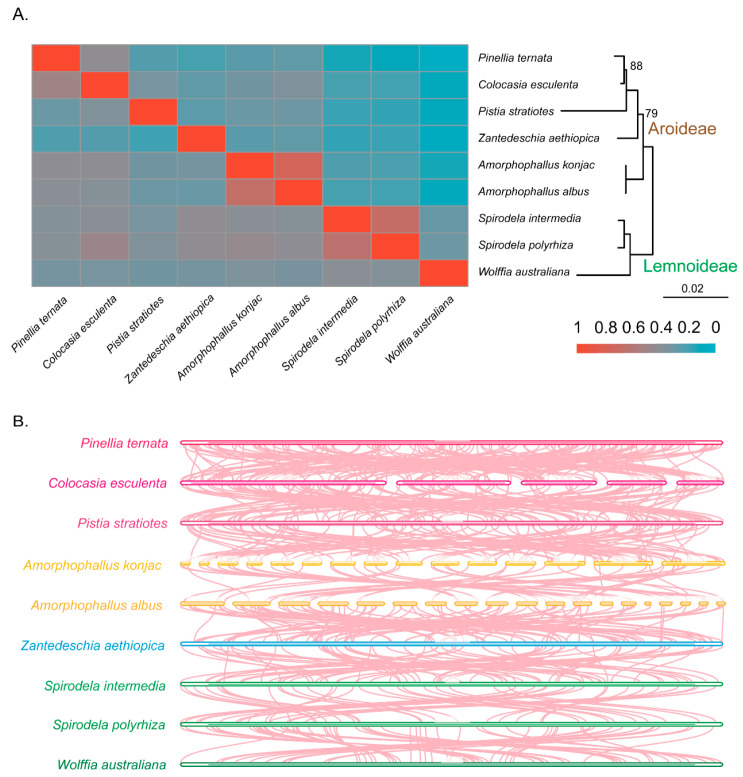
Analysis results of mitochondrial genomic sequences. (**A**) Shared DNA sequence; (**B**) the collinearity between two species.

Species within the genera *Spirodela* and *Amorphophallus* exhibited the highest proportion of shared mitochondrial DNA sequences (Figure 5A, Table S7). *C. esculenta* and *P. ternata* also showed substantial sequence sharing, with approximately 40% of their mitogenomes in common. In contrast, *W. australiana*, which possesses the smallest mitogenome among the sampled taxa, shared the greatest proportion of its sequences with *Spirodela*, while its shared DNA with *P. ternata* was less than 6%. Notably, *W. australiana* shared only about 10% of its mitogenome with the other species, highlighting its highly reduced and divergent mitogenome. Overall, the extent of sequence sharing among Araceae mitogenomes was broadly consistent with phylogenetic relatedness, with closer taxa exhibiting greater overlap. In addition, our collinearity analyses revealed extensive genomic rearrangements across species (Figure 5B), underscoring the dynamic nature of Araceae mitochondrial genome organization.

### 3.4. Selection Pressure Dynamics Across Araceae mitogenomes

**Figure 6:**
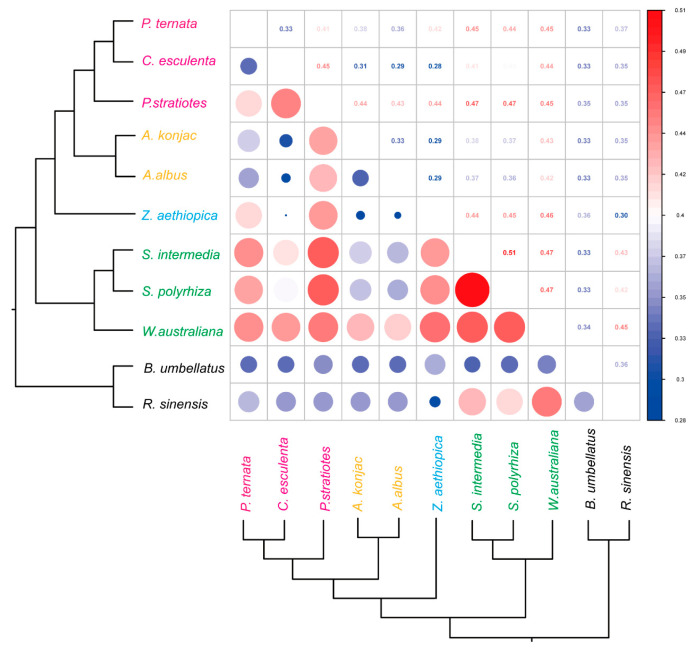
Pairwise selective pressure analysis of Araceae mitochondrial genomes. Upper triangle: *ω*-value matrix; Lower triangle: corresponding heatmap.

Because the Araceae comprises both aquatic and terrestrial taxa, we examined whether differences in habitat might impose distinct selective pressures on the mitogenome. We compared the selection pressures acting on respiration-related PCGs across Araceae species (Figure 6). All pairwise *d_N_*/*d_S_* values were below 1, and most were below 0.5, consistent with strong purifying selection. The only exception was the comparison between *S. intermedia* and *S. polyrhiza*, which showed a slightly elevated value of 0.51. These results indicate that mitochondrial PCGs in Araceae are generally conserved and primarily constrained by purifying selection, regardless of habitat type. Importantly, the absence of distinct selective patterns between aquatic and terrestrial taxa suggests that habitat shifts within Araceae did not substantially alter the evolutionary constraints on mitochondrial genes associated with respiration.

### 3.5. RNA Editing Site in Araceae mitogenomes

We analyzed RNA-seq data from five representative species in Araceae—*C. esculenta*, *P. stratiotes*, *A. konjac*, *S. polyrhiza*, and *W. australiana*—to identify RNA editing sites in their mitogenomes. For *A. konjac*, *C. esculenta*, and *S. polyrhiza*, datasets covering multiple developmental stages or tissues were included, thereby providing a more comprehensive basis for detecting editing sites.

Our results revealed marked variation in the number of RNA editing sites among the five species (Table 2). The highest number of editing sites was detected in *W. australiana*, with a total of 772 sites, of which 533 were located in coding regions and 239 in non-coding regions. *C. esculenta* ranked second, harboring 768 sites (508 in coding regions and 260 in non-coding regions). In contrast, *A. konjac* and *S. polyrhiza* exhibited a moderate number of editing sites (~400 sites each), whereas *P. stratiotes* had the lowest count, with only 363 sites (301 in coding regions and 62 in non-coding regions).

**Figure 7:**
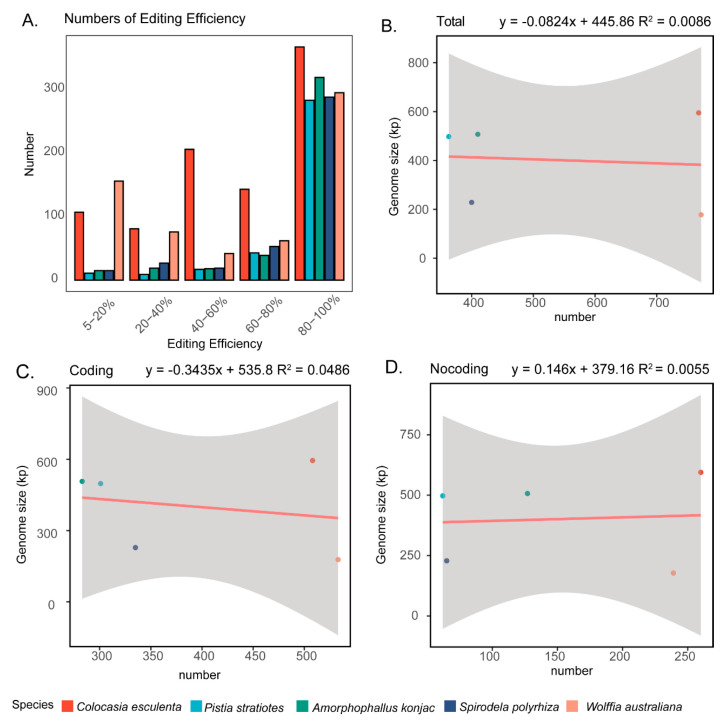
RNA editing sites in Araceae mitogenomes. (**A**) Editing efficiency of RNA editing sites in the five analyzed Araceae species. (**B**–**D**) Correlation analyses between the number of RNA editing sites with genome size, the length of coding region and noncoding regions.

In terms of editing efficiency, we found that across all five species, the vast majority of RNA editing sites were high-efficiency edits, with editing levels reaching 80–100% (Figure 7A). This pattern suggests that most mitochondrial RNA editing events are strongly favored and likely essential for maintaining proper mitochondrial function. Interestingly, despite the large differences in editing site numbers, we observed no significant correlation between the number of editing sites and mitogenome size, coding region length, or non-coding region length (Figure 7B–D). Collectively, these findings highlight substantial interspecific variation in the extent of RNA editing within Araceae mitogenomes, while also indicating that editing efficiency remains consistently high across species.

## 4. Discussion

### 4.1. Extensive Structural and Size Variation in Araceae mitogenomes

Comparative analysis of the mitogenomes from nine representative Araceae species uncovered remarkable heterogeneity in both size and structural organization, reflecting the well-documented plasticity of plant mitogenomes [43,44]. Genome size exhibited more than a fourfold difference, ranging from ~178 kb in the aquatic *W. australiana* to ~877 kb in the terrestrial *P. ternata* [18]. Notably, *W. australiana* harbors the smallest known mitogenome among photoautotrophic angiosperms [2], underscoring extreme genome reduction within this aquatic lineage. By contrast, five Araceae species displayed mitogenome sizes exceeding 500 kb, suggesting that expansions and contractions occurred independently within different ecological contexts [45]. Despite these size differences, the total length of coding regions was relatively conserved across the nine species, indicating that intergenic regions, rather than coding region, represent the primary driver of size variation, consisted with the previous studies [45,46]. Within Araceae, the largest contributors to genome expansion were sequences of unknown origin, this observation is consistent with widespread horizontal DNA transfer into angiosperm mitogenomes [7,46]. The second major contributor to size heterogeneity was expansion or loss of repetitive sequences, which are well known to promote recombination-mediated structural variation [45,47]. By contrast, plastid-derived DNA (MTPTs) played a comparatively minor role, echoing patterns reported in other angiosperms such as Apocynaceae and *Cycas taitungensis* C. F. Shen, K. D. Hill, C. H. Tsou & C. J. Chen [48,49]. Additionally, synteny and homologous sequence analyses further demonstrated extensive rearrangement and a paucity of collinear blocks among Araceae mitogenomes, in line with the highly dynamic nature of plant mitochondrial architecture [50,51,52].

Structurally, Araceae mitogenomes encompass nearly the full spectrum of mitochondrial configurations known in angiosperms [3,4]. For instance, *P. ternata* and *W. australiana* maintain relatively simple, single-circular chromosomes, while *Amorphophallus* species exhibit a strikingly multipartite organization with up to 19 circular chromosomes [17]. Similar extreme fragmentation has been described in *Silene*, *Lophophytum* and *Rhopalocnemis* [53,54,55], with repeat-mediated homologous recombination widely considered the mechanistic basis [56,57]. Our observation of abundant repeats in *Amorphophallus* strongly supports this model. Interestingly, among the species analyzed, aquatic Araceae species tend to have smaller and structurally simpler mitogenomes than their terrestrial relatives, although broader sampling is needed to determine whether this pattern is general across the family. This pattern parallels mitogenome streamlining reported in seagrasses (*Zostera marina* L. and *Phyllospadix iwatensis* Makino) [20,58], suggesting convergent selective pressures in aquatic environments. Possible drivers include reduced horizontal DNA acquisition due to ecological isolation, or metabolic streamlining linked to hypoxic stress and altered respiration demands in aquatic habitats [59]. Such habitat-associated signatures highlight that while Araceae mitogenomes conform to the general paradigm of structural plasticity, they also reveal ecological relevance in shaping the balance between expansion and reduction. Collectively, these findings reinforce that Araceae mitogenomes are shaped by a complex interplay of DNA acquisition, repeat-mediated rearrangements, and habitat-driven constraints. They both conform to and extend our understanding of angiosperm mitogenome evolution by providing a compelling case of structural diversity linked with ecological adaptation.

### 4.2. Functional Conservation and Lineage-Specific Divergence

Despite the striking variation in mitogenome size and structural organization within the Araceae, their functional gene repertoire remains remarkably conserved, consisted with patterns reported across seed plants [60,61,62]. All nine analyzed species retain the canonical set of PCGs essential for oxidative phosphorylation, including subunits of complexes I–V of the respiratory electron transport chain. Gene loss is restricted primarily to ribosomal PCGs, which have undergone repeated and independent transfers to the nuclear genome throughout seed plant evolution [63,64]. This pattern suggests that, while structural and size evolution in mitogenomes is highly dynamic, the retention of respiratory genes is under strong evolutionary constraint, reflecting their indispensable role in energy metabolism [65,66]. Molecular evolutionary analyses further support this functional conservation. Core PCGs, particularly those involved in respiratory electron transport, are consistently subject to strong purifying selection in both aquatic and terrestrial lineages. Although aquatic species tend to exhibit smaller and more compact mitogenomes, this reduction does not correspond to a detectable relaxation of selective pressure on core respiratory genes. This mirrors findings in diverse angiosperms, including *Gossypium* and *Fragaria* [51,62,67], where respiratory genes exhibit extremely low nonsynonymous substitution rates, underscoring their functional indispensability. However, this apparent association between aquatic habitat and reduced mitogenome size may be confounded by phylogeny, as all sampled aquatic taxa belong to the Lemnoideae subfamily. Thus, size reduction may also reflect lineage-specific genomic evolution. Accordingly, our interpretation remains cautious, recognizing that the observed patterns may arise from both ecological transitions and shared evolutionary history. By contrast, ribosomal PCGs exhibit relaxed selection or frequent loss, highlighting lineage-specific divergence in non-core functional components.

RNA editing represents another layer of post-transcriptional regulation that contributes to functional stability despite underlying sequence divergence [68]. Our comparative analysis revealed substantial variation in the number of predicted editing sites across Araceae species, ranging from 363 in *P. stratiotes* to 772 in *W. australiana*. Such lineage-specific differences are consistent with patterns reported in seed plants [22,69,70], where RNA editing site number varies considerably among species even when coding regions remain conserved. Importantly, the majority of RNA editing sites in Araceae exhibit high editing efficiency (>80%), paralleling patterns across angiosperms [22,71]. This suggests that RNA editing plays a crucial role in maintaining conserved amino acid sequences of respiratory proteins, buffering functional constraints against underlying nucleotide divergence. Interestingly, the number of RNA editing sites was independent of overall mitogenome size or structural complexity. Moreover, the number of RNA editing sites appears independent of both habitat type and mitogenome architecture, further indicating that regulatory dynamics governing RNA editing are shaped primarily by lineage-specific factors rather than ecological context. Similar independence between genome architecture and editing site abundance has been demonstrated in *Silene* and *Broussonetia* [71,72]. This decoupling indicates that the evolutionary trajectory of RNA editing sites is shaped more by lineage-specific regulatory dynamics than by mitogenome expansion or contraction [70]. One possible explanation is that the origin and persistence of RNA editing sites are influenced by nuclear-encoded RNA editing factors [68], which coevolve with mitochondrial genes. Collectively, these findings highlight that although ecological transitions may coincide with structural modifications, functional conservation of respiratory genes and post-transcriptional mechanisms is largely maintained across habitats. Therefore, the observed mitogenome reduction in aquatic species should be interpreted in light of both ecological adaptation and phylogenetic constraint.

## 5. Conclusions

In this study, we assembled and compared nine mitogenomes from Araceae, including four newly generated and five previously published species, to investigate patterns of structural diversity and functional conservation. Our analyses revealed striking heterogeneity in mitogenome architecture, with genome sizes differing more than fourfold and structures ranging from a single circular chromosome to highly multi-chromosomes. Despite this remarkable structural plasticity, the core repertoire of PCGs involved in respiration is conserved, and strong purifying selection acts across both aquatic and terrestrial lineages. RNA editing was abundant and highly efficient, yet its abundance showed no relationship to genome size, underscoring the independence of editing dynamics from structural variation. Collectively, these results underscore the dual nature of mitogenome evolution in Araceae: structural components are dynamic and lineage-specific, while core functional genes remain under stringent evolutionary constraint. By providing new genomic resources and comparative insights, this study establishes Araceae as a powerful model for exploring the balance between genomic plasticity and functional conservation in plant mitogenome evolution and offers a foundation for future investigations that integrate ecological, physiological, and molecular perspectives.

## Figures and Tables

**Table 1:** Characterization of mitogenomes sampled in this study.

Species	Accession	Habitat	Size (bp)	No. of Contigs	Gene			Introns		
					PCGs ^1^	tRNA	rRNA	Cis	Trans	Total
*Pinellia ternata*	NC_081910.1	terrestrial	876,608	1	37	17 (21)	3	18	6	24
*Colocasia esculenta*	PP389238-PP389242	terrestrial	594,811	5	36	17 (18)	3	19	5	24
*Pistia stratiotes*	contig1	emergent	497,568	1	35	19 (22)	3	18	6	24
*Amorphophallus albus*	OM066869-OM066887	terrestrial	537,044	19	36	20 (21)	3	19	5	24
*Amorphophallus konjac*	contig1-contig15	terrestrial	507,063	15	36 (41)	16	3	19	5	24
*Zantedeschia aethiopica*	NC_073008.1	terrestrial	675,575	1	34	19 (23)	3	18	5	23
*Spirodela intermedia*	contig1	emergent	256,603	1	36	20 (24)	3	18	5	23
*Spirodela polyrhiza*	NC_017840	emergent	228,493	1	36	18 (20)	3	18	5	23
*Wolffia australiana*	contig1	emergent	177,872	1	36	18 (21)	3	18	5	23
*Ruppia sinensis*	NC_088727.1	marine	256,174	1	28	12 (15)	3 (4)	15	6	21
*Butomus umbellatus*	KC208619.1	emergent	450,826	1	29 (34)	9 (10)	3	17	5	22

^1^ The number in brackets indicates the total number of genes including duplicated genes. PCGs: protein-coding genes. Cis indicates *cis*-spliced intron, while trans indicates *trans*-spliced intron.

**Table 2:** Overview of the RNA editing sites in Araceae.

		*Colocasia esculenta*	*Pistia stratiotes*	*Amorphophallus konjac*	*Spirodela polyrhiza*	*Wolffia australiana*
Total		768	363	410	400	772
Coding		508	301	283	335	533
	1st	152	96	98	91	158
	Non-silent	145	93	91	88	149
	silent	7	3	7	3	9
	2nd	300	187	162	202	285
	Non-silent	297	186	161	201	285
	silent	3	1	1	1	0
	3rd	56	18	23	42	90
	Non-silent	0	0	0	0	0
	silent	56	18	23	42	90
Non-coding		260	62	127	65	239
	intron	28	13	23	11	26
	rRNA	1	1	2	0	0
	tRNA	0	0	0	0	0
	intergenic	231	48	102	54	213

## Data Availability

The genomic resources generated in this study include four newly assembled mitogenomes and five re-annotated previously published Araceae mitogenomes. All assemblies and annotations are deposited in Figshare and can be accessed at https://figshare.com/s/af6c957a7ce117e9dd97, accessed on 25 September 2025.

## Supplementary Materials

The following supporting information can be downloaded at: https://www.mdpi.com/article/10.3390/genes16101241/s1, Table S1. Sequencing data used in this study [73,74,75,76,77]; Table S2. Organelle genomes included in this study; Table S3. Protein-coding gene content in sampled mitogenomes; Table S4. tRNA content in sampled mitogenomes; Table S5. Intron content in sampled mitogenomes; Table S6. Mitogenome composition and ratio in sampled mitogenomes; Table S7. Length and ratio of shared DNA in sampled mitogenomes.

## Abbreviations

The following abbreviations are used in this manuscript:

mitogenome	mitochondrial genome
PCGs	protein-coding genes
MTPTs	mitochondrial plastid DNA transfers
